# Quantitative assessment of polymorphisms in *H19* lncRNA and cancer risk: a meta-analysis of 13,392 cases and 18,893 controls

**DOI:** 10.18632/oncotarget.12530

**Published:** 2016-10-08

**Authors:** Minjie Chu, Weiyan Yuan, Shuangshuang Wu, Zhiquan Wang, Liping Mao, Tian Tian, Yihua Lu, Bowen Zhu, Yue Yang, Bin Wang, Haiquan Gao, Liying Jiang, Xun Zhuang

**Affiliations:** ^1^ Department of Epidemiology and Biostatistics, School of Public Health, Nantong University, Nantong, Jiangsu, China; ^2^ Department of Gastroenterology, Affiliated Hospital of Nantong University, Nantong, Jiangsu, China; ^3^ Department of Geriatrics, The First Affiliated Hospital of Nanjing Medical University, Nanjing, Jiangsu, China; ^4^ Center for Disease Control and Prevention of Nantong, Nantong, Jiangsu, China; ^5^ Department of Oncology, the Sixth People's Hospital of Nantong, Nantong, Jiangsu, China; ^6^ Nantong Prison Hospital, Nantong, Jiangsu, China

**Keywords:** H19, polymorphism, cancer and meta-analysis

## Abstract

*H19* refers to a long non-coding RNA (lncRNA) that functions as an oncogenic molecule in different cancer cells. Genetic variants of *H19* may affect the activity of certain regulatory factors, which subsequently regulate the aberrant expression of *H19*. This feedback loop might be one of the underlying mechanisms influencing tumour susceptibility and prognosis. Although there have been several recent studies that examined possible links between polymorphisms in *H19* and cancer risk, the results have been inconclusive. Thus, we performed a meta-analysis to estimate the associations between *H19* polymorphisms (rs2107425, rs2839698 and rs217727) and cancer risk. Ten studies comprising 13,392 cases and 18,893 controls were included in the study. Overall, the variant T allele of rs2107425 correlated with a significantly decreased risk of developing cancer (dominant model: OR = 0.86; 95% CI = 0.76–0.98). In addition, a marginally significant association between the rs2839698 and cancer risk was observed (dominant model: OR = 1.09; 95% CI = 0.99–1.20). After stratification for ethnicity, it became apparent that Asians with the variant A allele of rs2839698 exhibited a significantly higher risk of developing cancer (dominant model: OR = 1.11; 95% CI = 1.01–1.23). Interestingly, the rs2839698 variant was also significant associated with an increased risk of cancers of the digestive system (dominant model: OR = 1.23; 95% CI = 1.08–1.41). These findings provided evidence that *H19* rs2107425 may modify general cancer susceptibility, while rs2839698 may modify cancer susceptibility based on ethnicity and type. Further experimental studies to evaluate the limits of this hypothesis are warranted, and future functional studies are required to clarify the possible mechanisms.

## INTRODUCTION

Cancer has long been a major public health problem. Currently, it is the leading cause of morbidity and mortality worldwide. In 2016, it is estimated that 1,685,210 new cancer cases and 595,690 cancer deaths will occur in the United States [1], while an estimated 4,292,000 new cancer cases and 2,814,000 cancer deaths were projected in China in 2015 [2]. Various factors contribute to cancer's development, with environmental and genetic factors being the most common. In particular, the emergence of high-throughput RNA sequencing (RNA-Seq) technologies have provided a revolutionary means for systematically analysing the role of non-coding genomic transcripts in regulating gene expression, and by extension their impact on disease development and progression. Among these transcripts, long non-coding RNAs (lncRNAs) are emerging as significant regulators in tumourigenesis and progressions [3, 4], and numerous lncRNAs have been identified in multiple cancer transcriptomes [5]. One such lncRNA is the imprinted maternally expressed, non-protein coding transcript *H19*. The relationship between an aberrant expression of *H19* and cancer prognosis has been explored by many researchers. Furthermore, a recent meta-analyses performed by Chen et al. has demonstrated that high levels of *H19* expression may serve as a predictive indicator of poor prognoses in multiple cancers. Meta-analysis has also revealed that a high expression of *H19* is significantly related to lymph node metastasis, another influence on cancer prognoses [6].

Recently, genome-wide association study (GWAS) has identified a significant association between the single nucleotide polymorphism (SNP) rs2107425 and breast cancer risk [7]. SNP rs2107425 is located approximately 2 kb upstream of *H19* lncRNA. In 2012, Riaz et al. analysed mRNA expression of the gene *H19* most likely to be closely located to SNP rs2107425 in a subset of 1,401 primary breast tumours. However, no significant difference between the respective three genotype groups of rs2107425 and *H19* mRNA expression was observed [8]. These results indicate that the SNP rs2107425 is unlikely itself to be a causative agent of breast cancer and that a more thorough evaluation of variations in the associated *H19* gene region is warranted. As expected, the associations of *H19* polymorphisms with cancer sensitivities have attracted much interest, with particular focus on *H19* rs2839698 and rs217727. Based on the Encyclopedia of DNA Elements (*ENCODE*) DNase I hypersensitive site (DHS) sequencing data set, we found the two *H19* SNPs (rs2839698 and rs217727) are within open chromatin regions associated with gene regulatory elements, indicating that both of the SNPs may affect the binding of transcription factors. ChIP-Seq data from the ENCODE project further demonstrates that rs217727 is located in a region that may influence the binding of numerous transcription factors. These results show that it is biologically conceivable for the SNPs (rs2839698 and rs217727) in *H19* to be potential causal variants that regulate the expression of *H19* and further affect cancer development and progression.

As expected, the association of two SNPs (rs2839698 and rs217727) together with rs2107425 in *H19* with cancers susceptibility has attracted much interest in subsequent research [9–18]. Unfortunately, the research exploring this association has not been able to reach a consensus. For instance, a previous study reported that the variant genotype of rs2839698 was definitely associated with increased risk for colorectal and gastric cancers in the Chinese population [9, 11]. However, the rs2839698 polymorphism exhibited the opposite associations for cancer risk in Caucasians (a population in the Netherlands) [14]. Thus, we performed a meta-analysis using currently published data to more precisely characterize the associations of rs2107425, rs2839698 and rs217727 in *H19* lncRNA with cancer risks.

## RESULTS

### Characteristics of the published studies

**Figure 1:**
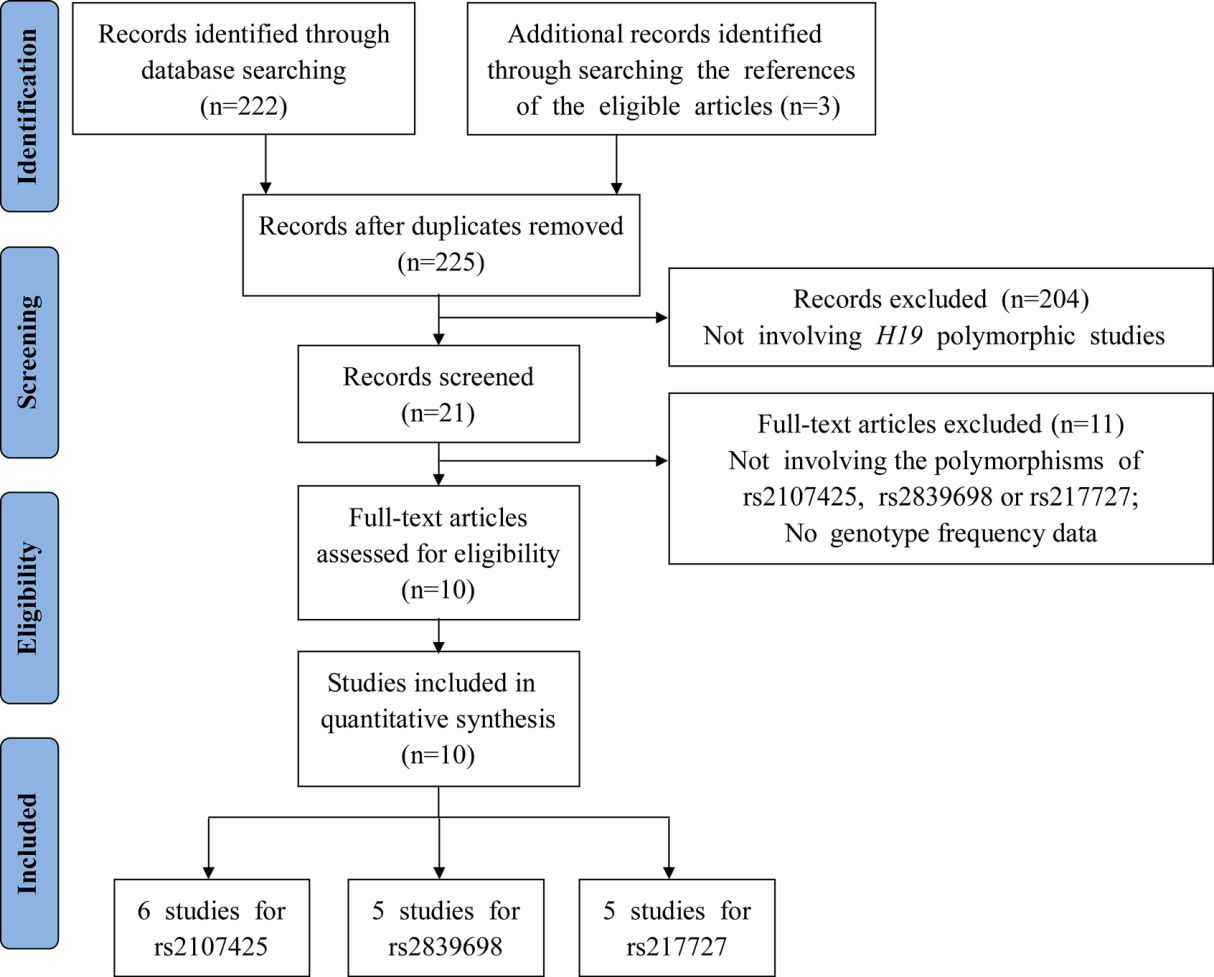
Flow diagram of the study selection process

Following the application of strict screening criteria, 10 articles evaluating a total of 13,392 cases and 18,893 controls concerning gastric, bladder, colorectal, breast, ovarian and lung cancers were ultimately included in our quantitative analysis (Figure 1). The general characteristics of the included studies are listed in Table 1. Among these studies, five studies were carried out among Asian populations and five studies were carried out among Caucasian populations. Three articles reported the effects of *H19* polymorphisms in breast cancer, two reported in bladder cancer, two in ovarian cancer, one in gastric cancer, one in lung cancer and one in colorectal cancer. Among the studies that explored the relationships between *H19* SNPs with cancer risk, one focused on 3 SNPs (rs2107425, rs2839698 and rs217727), and four focused on 2 SNPs, while the other five focused on only one SNP. Genotyping was performed using TaqMan in 5 studies, Sequenom in 3 studies, created restriction site PCR (CRS-RFLP) in 1 study, GoldenGate assay in 1 study and PCR-RFLP in 1 study. In addition, there was no evidence to prove that genotype frequencies among the controls deviated from those expected under the Hardy-Weinberg equilibrium (HWE) for each SNP studied. The distributions of genotypes and alleles of *H19* polymorphisms (rs2107425, rs2839698 and rs217727) for each individual study are listed in Supplementary Tables S2–S4.

**Table 1:** Characteristics of the studies included in the meta-analysis

First Author	Year	Country	Ethnicity	Type of cancer	Case/Control	Source of controls	Platform	Genotyped SNPs
Hua	2016	China	Asian	bladder cancer	1049/1399	Hospital-based	TaqMan	rs217727, rs2839698
Li	2016	China	Asian	colorectal cancer	1147/1203	Population-based	TaqMan	rs217727, rs2839698
Xia	2016	China	Asian	breast cancer	464/467	Population-based	CRS-RFLP^a^	rs217727
Gong	2016	China	Asian	lung cancer	498/213	Hospital-based	Sequenom	rs2839698, rs2107425
Yang	2015	China	Asian	gastric cancer	500/500	Hospital-based	TaqMan	rs217727, rs2839698
Butt	2012	Sweden	Caucasian	breast cancer	728/1448	Population-based	Sequenom	rs2107425
Barnholtz-Sloan	2010	USA	Caucasian	breast cancer	1972/1776	Population-based	GoldenGate assay	rs2107425
Quaye	2009	Multinational^b^	Caucasian	ovarian cancer	1491/3145	Population-based	Taqman	rs2107425
Song	2009	Multinational^c^	Caucasian	ovarian cancer	5366/8538	Population-based	Taqman + Sequenom	rs2107425
Verhaegh	2008	The Netherlands	Caucasian	bladder cancer	177/204	Population-based	PCR-RFLP	rs217727, rs2839698, rs2107425

a created restriction site PCR.

b including UK, Denmark and USA.

c including European countries, USA and Australia.

### Quantitative synthesis

**Figure 2:**
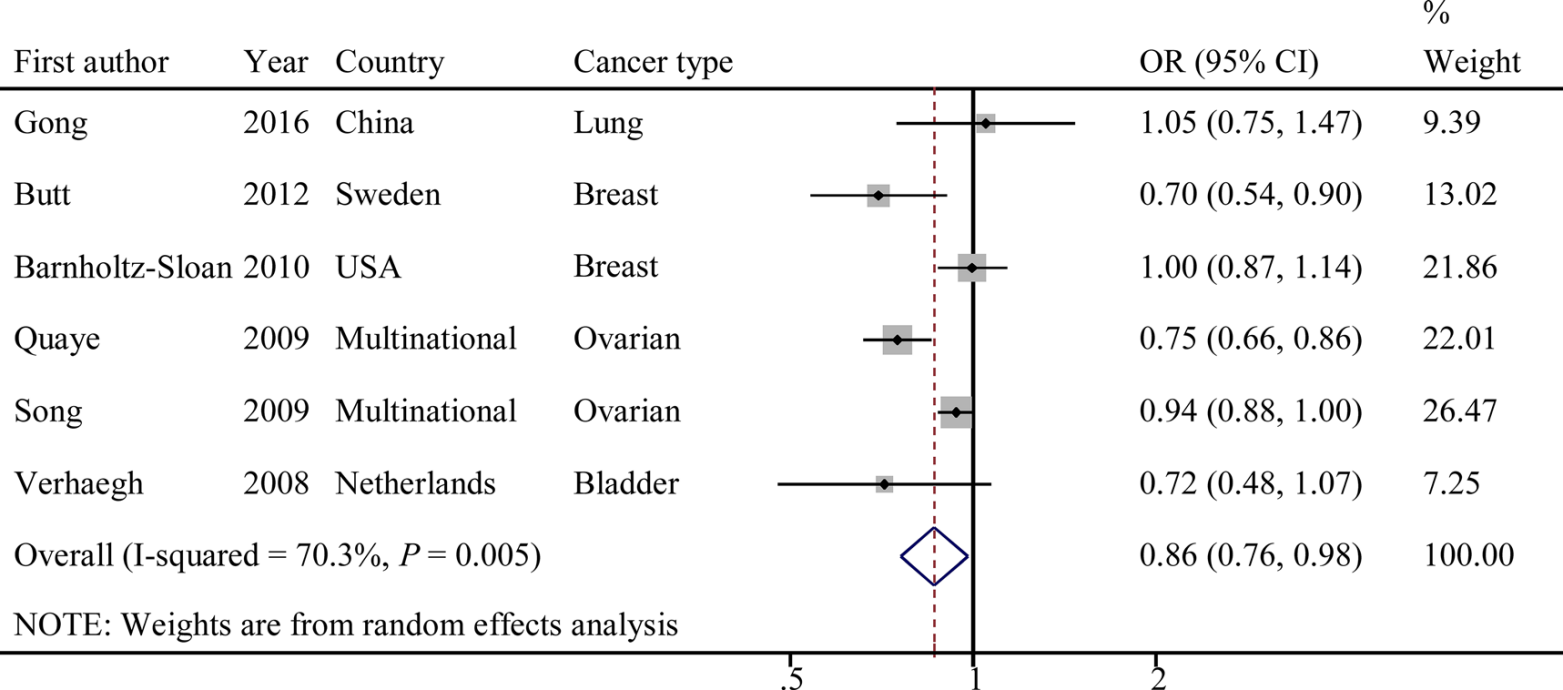
Forest plot of OR with 95%CI for the *H19* rs2107425 with cancer risk under dominant model

Evaluations of the associations of rs2107425 with cancer risks are presented in Table 2. The variant T allele of rs2107425 was correlated with a significantly decreased risk of developing cancer (dominant model: OR = 0.86; 95% CI = 0.76–0.98, *P* = 0.005 for the heterogeneity test, *I^2^* = 70.3%; Figure 2). The results of other tested models are listed in Table 2. Next, we evaluated the effect of the rs2107425 polymorphism on cancer risk among the subgroups (Table 2). In the stratified analyses, associations between rs2107425 and cancer risk were still significant among Caucasians (dominant model: OR = 0.84; 95% CI = 0.74–0.97; *P* = 0.003 for the heterogeneity test, *I^2^* = 75.0%), in studies with population-based controls (dominant model: OR = 0.84; 95% CI = 0.74–0.97; *P* = 0.003 for the heterogeneity test, *I^2^* = 75.0%) and in studies with case sample size ≥ 500 (dominant model: OR = 0.86; 95% CI = 0.74−0.99; *P* = 0.002 for the heterogeneity test, *I^2^* = 79.7%).

**Table 2:** Summary ORs of the *H19* rs2107425 polymorphism and cancer risk

Variables	Studies	CT versus CC			TT versus CC			Dominant model		
		OR(95%CI)	*P*^a^	*I^2^*	OR (95%CI)	*P*^a^	*I^2^*	OR(95%CI)	*P*^a^	*I^2^*
Total	6	**0.84 (0.73–0.97)**	0.002	73.6%	0.97 (0.89–1.06)	0.798	0.0%	**0.86 (0.76–0.98)**	0.005	70.3%
*Ethnicity*										
Asians	1	1.07 (0.75–1.52)			0.98 (0.59–1.65)			1.05 (0.75–1.47)		
Caucasians	5	**0.82 (0.71–0.96)**	0.001	77.5%	0.97 (0.89–1.06)	0.671	0.0%	**0.84 (0.74–0.97)**	0.003	75.0%
*Cancer type*										
breast cancer	2	0.84 (0.58–1.23)	0.014	83.4%	0.94 (0.79–1.12)	0.329	0.0%	0.85 (0.60–1.20)	0.016	82.6%
ovarian cancer	2	0.82 (0.65–1.04)	0.002	89.2%	0.98 (0.89–1.09)	0.268	18.4%	0.84 (0.68–1.05)	0.003	88.6%
lung cancer	1	1.07 (0.75–1.52)			0.98(0.59–1.65)			1.05 (0.75–1.47)		
bladder cancer	1	0.66 (0.43–1.01)			1.02 (0.51–2.03)			0.72 (0.48–1.07)		
*Source of controls*										
Population-based	5	**0.82 (0.71–0.96)**	0.001	77.5%	0.97 (0.89–1.06)	0.671	0.0%	**0.84 (0.74–0.97)**	0.003	75.0%
Hospital-based	1	1.07 (0.75–1.52)			0.98 (0.59–1.65)			1.05 (0.75–1.47)		
*Case sample size*										
≥ 500	4	**0.84 (0.72–0.98)**	0.001	81.2%	0.97 (0.89–1.06)	0.506	0.0%	**0.86 (0.74–0.99)**	0.002	79.7%
< 500	2	0.88 (0.67–1.15)	0.085	66.30%	0.99 (0.66–1.51)	0.934	0.00%	0.90 (0.69–1.16)	0.154	50.90%

a *P* for heterogeneity (a random-effects model was used when the *P* value for heterogeneity test was < 0.05; otherwise, a fixed-effect model was used).

**Figure 3:**
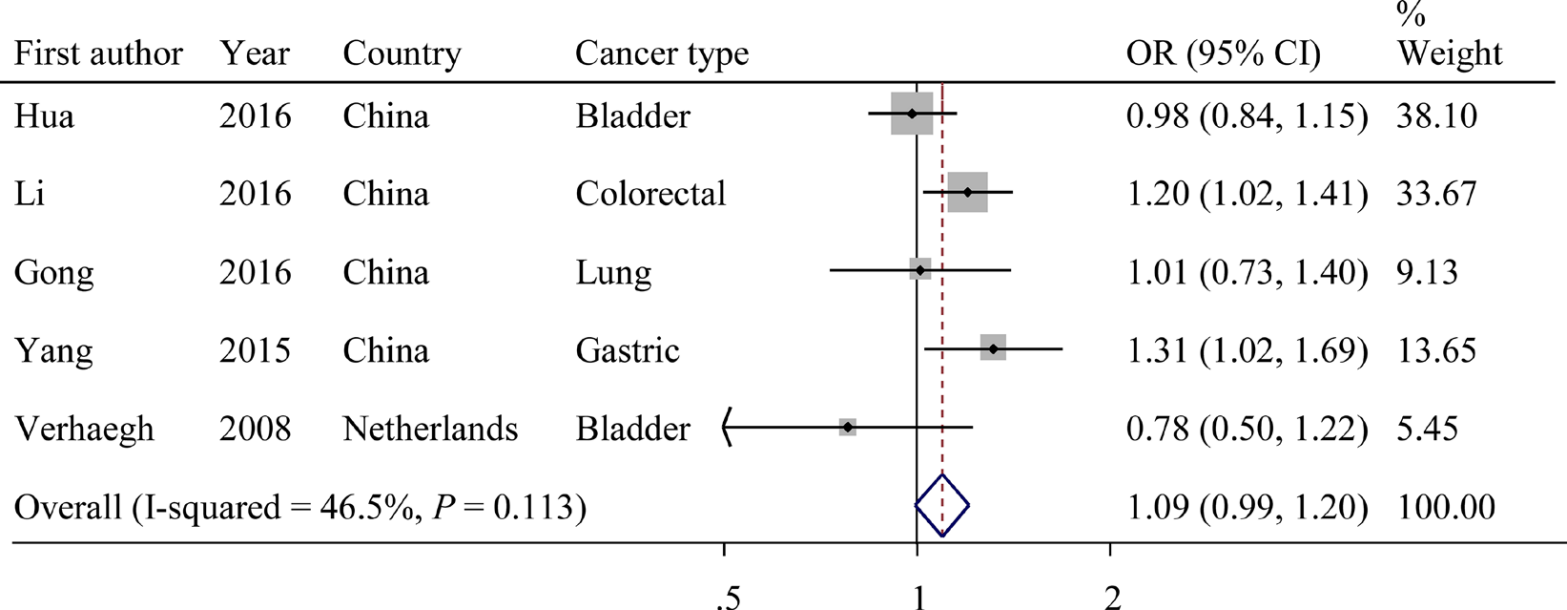
Forest plot of OR with 95%CI for the *H19* rs2839698 with cancer risk under dominant model

The evaluations of the associations of rs2839698 with cancer risks are presented in Table 3. The variant A allele exhibited a marginally significant association with cancer risk in the dominant model (OR = 1.09; 95% CI = 0.99−1.20, *P* = 0.113 for the heterogeneity test, *I^2^* = 46.5%; Figure 3). The results of other tested models are listed in Table 3.

**Table 3:** Summary ORs of the *H19* rs2839698 polymorphism and cancer risk

Variables	Studies	CT versus CC			TT versus CC			Dominant model		
		OR(95%CI)	*P*^a^	*I^2^*	OR (95%CI)	*P*^a^	*I^2^*	OR(95%CI)	*P*^a^	*I^2^*
Total	5	1.07 (0.96–1.18)	0.130	43.8%	1.15 (0.83–1.58)	0.019	66.1%	1.09 (0.99–1.20)	0.113	46.5%
*Ethnicity*										
Asians	4	1.09 (0.98–1.21)	0.396	0.0%	1.15 (0.78–1.69)	0.008	74.4%	**1.11 (1.01–1.23)**	0.158	42.3%
Caucasians	1	0.65 (0.40–1.06)			1.10 (0.63–1.92)			0.78 (0.50–1.22)		
*Cancer type*										
digestive system cancer ^b^	2	**1.17 (1.02–1.35)**	0.594	0.0%	**1.58 (1.22–2.05)**	0.840	0.0%	**1.23 (1.08–1.41)**	0.546	0.0%
bladder cancer	2	0.94 (0.80–1.10)	0.123	58.0%	1.03 (0.79–1.36)	0.807	0.0%	0.96 (0.82–1.11)	0.340	0.0%
lung cancer	1	1.15 (0.81–1.63)			0.60 (0.35–1.04)			1.01 (0.73–1.40)		
*Source of controls*										
Population-based	2	0.91 (0.53–1.55)	0.032	78.2%	**1.43 (1.08–1.88)**	0.290	10.9%	1.14 (0.98–1.33)	0.076	68.2%
Hospital-based	3	1.06 (0.93–1.21)	0.284	20.6%	1.02 (0.62–1.67)	0.019	74.7%	1.06 (0.94–1.20)	0.149	47.6%
*Case sample size*										
≥ 500	3	1.09 (0.97–1.21)	0.237	30.5%	**1.32 (1.08–1.61)**	0.096	57.3%	**1.12 (1.01–1.24)**	0.089	58.7%
< 500	2	0.95 (0.72–1.25)	0.063	71.1%	0.81 (0.55–1.20)	0.133	55.7%	0.92 (0.71–1.20)	0.357	0.0%

a *P* for heterogeneity (a random-effects model was used when the *P* value for heterogeneity test was < 0.05; otherwise, a fixed-effect model was used).

Next, we evaluated the effect of the rs2839698 polymorphism on cancer risk among the subgroups (Table 3). In the stratified analyses, the rs2839698 SNP had significant association with increased cancer risk among Asians (Chinese; dominant model: OR = 1.11; 95% CI = 1.01–1.23; *P* = 0.158 for the heterogeneity test, *I^2^* = 42.3%). Beyond that, the rs2839698 variant exhibited a significant association with an increased risk of digestive system cancers (dominant model: OR = 1.23; 95% CI = 1.08–1.41; *P* = 0.546 for the heterogeneity test, *I^2^* = 0.0%). Interestingly, the variant A allele of rs2839698 was significantly associated with an increased risks of developing cancer among studies with a case sample size ≥ 500 (dominant model: OR = 1.12; 95% CI = 1.01−1.24; *P* = 0.089 for the heterogeneity test, *I^2^* = 58.7%).

**Figure 4:**
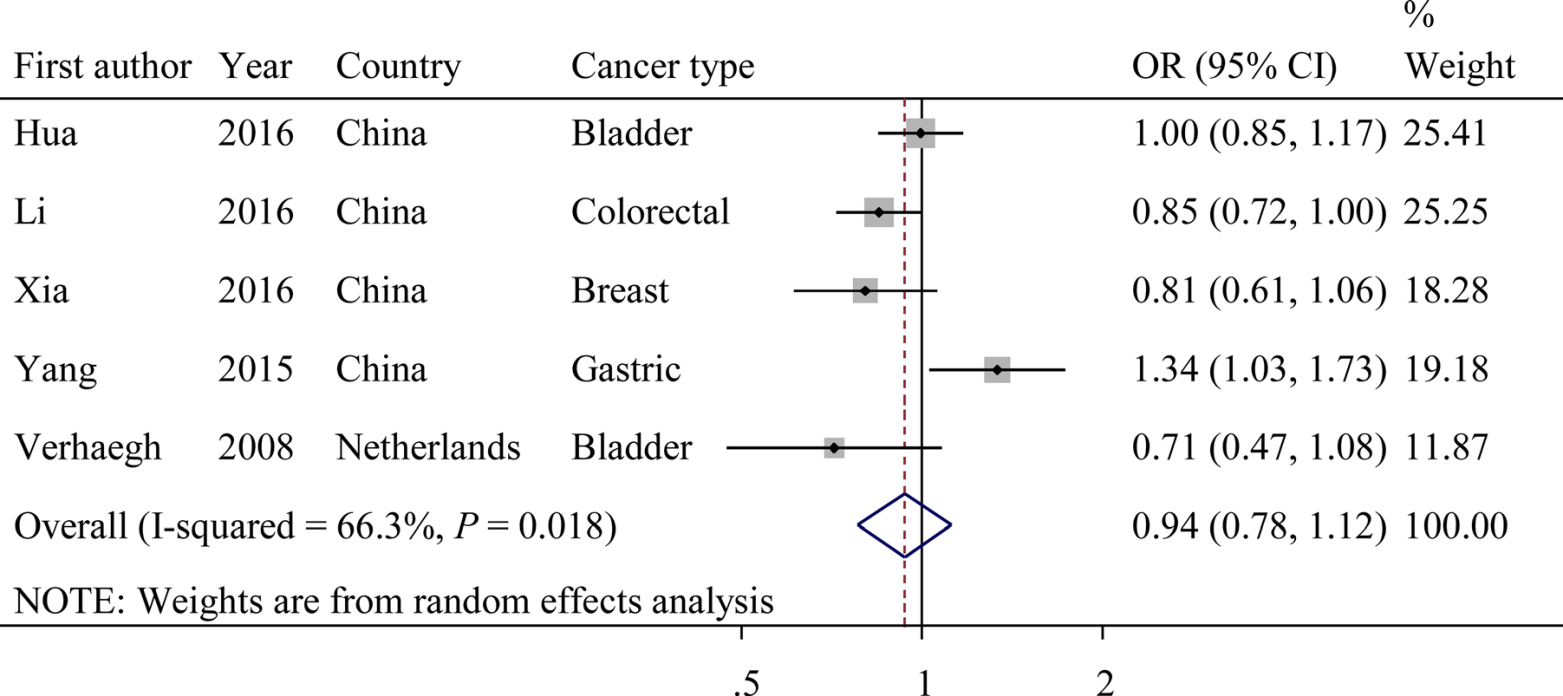
Forest plot of OR with 95%CI for the *H19* rs217727 with cancer risk under dominant model

The evaluations of the associations between SNP rs217727 and cancer risk are displayed in Figure 4. Overall, the A variant allele of rs217727 exhibited no significant association with cancer risks (dominant model: OR = 0.94; 95% CI = 0.78–1.12, *P* = 0.018 for the heterogeneity test, *I^2^* = 66.3%). The results of other tested models are listed in Table 4. In further stratified analyses, rs217727 SNP was significantly associated with decreased cancer risk among studies with population-based controls (dominant model: OR = 0.82; 95% CI = 0.72−0.94; *P* = 0.739 for the heterogeneity test, *I^2^* = 0.0%). Additionally, the variant A allele of rs217727 was significantly associated with a decreased risk of developing cancer among studies with a case sample size < 500 (dominant model: OR = 0.78; 95% CI = 0.62–0.98; *P* = 0.636 for the heterogeneity test, *I^2^* = 0.0%; Table 4).

**Table 4:** Summary ORs of the *H19* rs217727 polymorphism and cancer risk

Variables	Studies	GA versus GG			AA versus GG			Dominant model		
		OR(95%CI)	*P*^a^	*I^2^*	OR (95%CI)	*P*^a^	*I^2^*	OR(95%CI)	*P*^a^	*I^2^*
Total	5	0.88 (0.73–1.06)	0.022	65.0%	1.10 (0.83–1.48)	0.011	69.5%	0.94 (0.78–1.12)	0.018	66.3%
*Ethnicity*										
Asians	4	0.90 (0.73–1.11)	0.014	71.9%	1.16 (0.87–1.54)	0.012	72.7%	0.97 (0.80–1.17)	0.018	70.3%
Caucasians	1	0.74 (0.49–1.14)			0.45 (0.13–1.50)			0.71 (0.47–1.08)		
*Cancer type*										
digestive system cancer^b^	2	1.02 (0.71–1.47)	0.024	80.4%	1.16 (0.57–2.34)	0.002	89.3%	1.05 (0.67–1.64)	0.004	88.0%
bladder cancer	2	0.90 (0.77–1.06)	0.331	0.0%	1.20 (0.93–1.54)	0.100	63.0%	0.95 (0.82–1.11)	0.143	53.5%
breast cancer	1	0.64 (0.47–0.87)			1.11 (0.79–1.55)			0.81 (0.61–1.06)		
*Source of controls*										
Population-based	3	**0.79 (0.69–0.91)**	0.256	26.6%	0.90 (0.74–1.09)	0.192	39.4%	**0.82 (0.72–0.94)**	0.739	0.0%
Hospital-based	2	1.01 (0.88–1.17)	0.080	67.3%	**1.38 (1.11–1.71)**	0.221	33.3%	1.08 (0.94–1.24)	0.061	71.6%
*Case sample size*										
≥ 500	3	0.95 (0.85–1.06)	0.076	61.1%	1.18 (0.80–1.75)	0.004	81.8%	1.02 (0.81–1.28)	0.015	76.2%
< 500	2	**0.67 (0.53–0.86)**	0.571	0.0%	1.03 (0.75–1.42)	0.156	50.30%	**0.78 (0.62–0.98)**	0.636	0.0%

a *P* for heterogeneity (a random-effects model was used when the *P* value for heterogeneity test was < 0.05; otherwise, a fixed-effect model was used).

### Test of heterogeneity

For rs217727, significant heterogeneity was observed after the data were pooled (dominant model: *P* for heterogeneity = 0.018, *I^2^* = 66.3%). As shown in Table 4, when the subjects were stratified on the basis of ethnicity, heterogeneity remained in Asians (dominant model: *P* for heterogeneity = 0.018, *I^2^* = 70.3%). Additionally, in analyses of control sources, the heterogeneity disappeared in studies with population-based controls (dominant model: *P* for heterogeneity = 0.739, *I^2^* = 0.0%), as well as in hospital-based controls (dominant model: *P* for heterogeneity = 0.061, *I^2^* = 71.6%). Furthermore, heterogeneity disappeared in studies with a case sample size < 500 (dominant model: *P* for heterogeneity = 0.636, *I*^2^ = 0.0%). Nonetheless, heterogeneity was still present in studies with a case sample size ≥ 500 (dominant model: *P* for heterogeneity = 0.015, *I*^2^ = 76.2%).

### Sensitivity analysis

To test the stability of the rs217727 results, we conducted sensitivity analyses by sequentially removing each eligible study (Supplementary Table S5). A study by Yang et al. that focused on gastric cancer was the major contributor of heterogeneity in the dominant model (*I^2^* = 66.3%, *P* for heterogeneity = 0.018). After removing this study, heterogeneity was significantly reduced (*I^2^* = 19.90%, *P* for heterogeneity = 0.290). As expected, similar results were observed in other genetic models (i.e., GA versus GG and AA versus GG), indicating that Yang et al.'s study on gastric cancer markedly changed the pooled OR.

### Publication bias

We utilized funnel plots and Begg's test to evaluate potential publication biases in the selected literature. As illustrated in Supplementary Figures S1–S3, the shapes of the funnel plots were symmetrical. Moreover, a Begg's test provided further statistical evidence for the absence of publication bias (*P* = 0.57 for rs2107425, *P* = 1.00 for rs2839698, and *P* = 0.62 for rs217727).

## DISCUSSION

In the current study, we performed a meta-analysis by pooling 10 studies with totals of 13,392 cases and 18,893 controls. We demonstrated that the variant T allele of rs2107425 exhibited a significant decreased risk for developing cancer, and the A allele of rs2839698 was associated with a significant increased cancer risk in Asians, as well as a significant association with an increased risk for digestive system cancers. In contrast, the rs217727 variant allele exhibited no significant association with cancer risks.

Recently, multiple studies have reported the significant role of *H19* in tumourigenesis. As suggested by Chen et al. in 2016, *H19* may promote gastric cancer cell migration and invasion [19], while similar results were observed in oesophageal squamous cell carcinoma cells [20, 21]. Our study indicates that *H19* may act as an oncogene and predict poor prognosis. Furthermore, bioinformatic analysis has shown that rs2839698 in *H19* may change crucial folding structures and alter the targeted microRNAs [11]. Using the prediction of the miRNA-binding analysis website (http://bioinfo.life.hust.edu.cn/miRNASNP2/index.php), we found that rs2839698 polymorphism in *H19* 3′ UTR may result in the loss of hsa-miR-24-1-5p and hsa-miR-24-2-5p function. MiR-24 acts as a tumour suppressor and is low-expressed in various types of cancers, including colorectal, prostate and bladder cancer [22–24]. Thus, it is biologically conceivable that the loss of miR-24 function owing to a SNP rs2839698 in the 3′ UTR of *H19* may give rise to overexpression of *H19* and thereby promote proliferation, migration and invasion of some cancer cells. However, experimental studies to evaluate the limits of this hypothesis are warranted, and future functional studies are required to clarify the possible mechanisms.

In this meta-analysis, we identified a marginally significant relationship of rs2839698 SNP with overall cancer risk. According to a stratified analysis, SNP rs2839698 was definitely associated with an increased risk of developing cancer for Asians (mainly Chinese patients). By contrast, among a population in the Netherlands, the same variant genotype of rs2839698 exhibited no significant association with cancer risk, and the effect value was even in the opposite direction relative to previous studies of Chinese populations. There are several possible reasons for different results between Asians and Caucasians. First, the difference may have resulted from differences in the genetic backgrounds of the studied populations. For instance, based on the HapMap data (International HapMap Project), the A allele frequency of the rs2839698 SNP is 0.28 in Asian populations (CHB+JPT) and 0.55 in European populations (CEU). Second, differences may stem from the utilization of different genotyping methods including PCR-RFLP, CRS-RFLP, TaqMan and Sequenom. Third, when compared with Asian populations, the sample sizes of the Caucasian populations might not have been sufficiently large to reach a convincing conclusion concerning the association of the rs2839698 SNP with cancer risks. Additionally, the different types of cancers involved and random errors may also have been potential reasons for different findings between Asians and Caucasians.

The sensitivity analysis of rs217727 found that the result of pooling ORs was significantly changed once Yang et al.'s study was excluded. This change could be accounted for by the 500 mixed types of gastric cancer patients (221 cardia gastric cancer patients and 279 non-cardia gastric cancer patients) enrolled in Yang et al.'s study, in contrast to the stricter criteria used for recruiting patients into other studies. To account for this difference, we separated Yang et al.'s study into two parts (cardia gastric cancer and non-cardia gastric cancer), and re-performed the meta-analysis with each part considered separately. Interestingly, heterogeneity obviously decreased when Yang et al.'s study was confined to non-cardia gastric carcinoma (dominant model: *P* for heterogeneity = 0.062, *I^2^* = 55.4%). However, when Yang et al.'s study was confined to cardia gastric carcinoma patients, heterogeneity was still present (dominant model: *P* for heterogeneity = 0.043, *I^2^* = 59.3%). So these conclusions should be considered cautiously.

The strength of our meta-analysis stems from systematically reviewing the relationships between three *H19* polymorphisms (rs2107425, rs2839698 and rs217727) and cancer susceptibility for the first time. In addition, the well-designed search and selection methods significantly increased the statistical power of this meta-analysis. However, there are also some limitations that need to be addressed. First, significant heterogeneity between the studies was observed for the analyses of rs2107425 and rs217727. Among the 10 published studies contained in our meta-analysis, some studies were population-based, while others were hospital-based. Second, in some studies, detailed information (e.g., age, gender, smoking status, and alcohol consumption) was not provided, which further limited the stratification analyses. Moreover, if we had been able to acquire more detailed information, we would have been able to achieve more precise estimations by adjusting for other potential covariates. Finally, few studies were included in this meta-analysis, and this small sample size limits the power to detect the associations. Because the power of funnel plots and Begg's test of publication bias may also greatly constrain our analysis, our conclusions should be interpreted cautiously. Well-conducted studies with larger sample sizes are needed to further explore the cancer risks related to *H19* SNPs, especially in Caucasians.

## MATERIALS AND METHODS

### Identification and eligibility criteria of relevant studies

A comprehensive literature search of research published before July 31, 2016 was performed using PubMed and Web of Science using the following keywords: (“*H19*”), (“cancer”, “carcinoma”, “tumor”, “tumour”, or “neoplasm”) and (“polymorphism”, “variation”, “variant”, or “mutation”). Only available full-text articles written in English were included in this meta-analysis. The references in the retrieved articles were also reviewed for possible inclusion. Studies were included if they met the following eligibility criteria: (1) case-control studies focused on the relationship between *H19* polymorphisms and any type of cancer, (2) at least two articles for each studied *H19* polymorphism, and (3) available information concerning the genotype frequency of each included *H19* SNP (i.e., rs2107425, rs2839698 or rs217727). The exclusion criteria were as follows: (1) Studies that did not focus on cancer risk, (2) did not study *H19* SNPs (rs2107425, rs2839698 or rs217727), (3) did not report the relevant genotype frequency data, (4) were not published in English, or (5) non-human research was involved. Finally, a total of 10 articles containing 13,392 cases and 18,893 controls were included in this meta-analysis (Figure 1).

### Data extraction

Two investigators (M.C. and L.M.) independently extracted the data and agreed on the criteria and selections. Each article was mined for the following information: year of publication; name of the first author, ethnicity and country of origin; the type of cancer studied; the numbers of cases and controls; the source of controls; genotyping platform; and genotyped SNPs. We categorized ethnicities either as Caucasian or Asian.

### Quality assessments of the included studies

The methodological quality of each included study was evaluated using the Newcastle-Ottawa quality assessment scale (NOS). Using this method, each study was judged on standard criteria and subsequently categorized based on three factors: selection, comparability, and exposure. Summary scores ranging from 0 to 9 points were calculated, where higher scores indicate lower risks of bias (Supplementary Table S1).

### Statistical analysis

The risk of cancer associated with each *H19* polymorphism was estimated in each study using the odds ratio (OR) and its 95% confidence interval (95% CI). The between-study heterogeneity was examined with a chi-square-based *Q* statistic test, with *P* ≤ 0.05 considered as statistically significant. When heterogeneity between studies was absent we pooled the results using fixed-effect models. Otherwise, a random-effects model was chosen. Subsequently, we evaluated the risks of heterozygous and variant homozygous genotypes relative to the wild-type homozygous genotype, and then assessed the risks of the combined heterozygous as well as variant homozygous genotypes relative to the wild-type homozygous genotype while assuming the dominant effects of the variant allele. We performed a stratification analysis based on ethnicity (divided into Caucasians and Asians), cancer type, source of controls and case sample size. Funnel plots and Begg's test were utilized to evaluate publication bias. All analyses were performed using Stata software, version 12.0 (Stata Corporation, College Station, TX, and USA).

## CONCLUSIONS

This meta-analysis provided evidence that *H19* rs2107425 may modify general cancer susceptibility, while rs2839698 may modify cancer susceptibility based on ethnicity and type. Beyond these conclusions, we believe further studies that incorporate subjects from different ethnic backgrounds combined with re-sequencing the marked regions and functional evaluations are warranted.

## SUPPLEMENTARY MATERIALS


