# Supplementary material for: Quantitative assessment of polymorphisms in H19 lncRNA and cancer risk: a meta-analysis of 13,392 cases and 18,893 controls
Source: Oncotarget. 2016 Oct 8;7(48):78631–9. doi: 10.18632/oncotarget.12530 (PMC5346665; doi:10.18632/oncotarget.12530)
Supplement: Supplementary file 1 [file oncotarget-07-78631-s001.pdf]

Quantitative assessment of polymorphisms in *H19* lncRNA and cancer risk: a meta-analysis of 13,392 cases and 18,893 controls

Supplementary Materials

Supplementary Table S1: Newcastle-Ottawa quality assessment scale for each included study

Studies	Selection				Comparability		Exposure			Total quality score
	Case definition adequate	Representativeness of the cases	Selection of controls	Definition of controls	Adjustment for age	Adjustment for lifestyle/ traditionalrisk factors	Ascertainment of exposure	Uniform method of ascertainment	Non-responserate	
Hua	1	1	0	1	1	1	1	1	0	7
Li	1	1	1	1	1	1	1	1	1	9
Xia	1	1	1	1	1	1	1	1	1	9
Gong	1	1	0	1	1	0	1	1	0	6
Yang	1	1	0	1	1	1	1	1	1	8
Butt	1	1	1	1	1	1	1	1	0	8
Barnholtz-Sloan	1	1	1	1	1	0	1	1	0	7
Quaye	1	1	1	1	0	0	1	1	0	6
Song	1	1	1	1	1	0	1	1	0	7
Verhaegh	1	1	1	1	1	1	1	1	1	9

Supplementary Table S2: Distributions of the genotypes and alleles of the *H19* rs2107425 polymorphism

Number	First Author	Frequency distributions of the genotypes						HWE[a]
		Case			Control			
		CC N (%)	CT N (%)	TT N (%)	CC N (%)	CT N (%)	TT N (%)	
1	Gong	181 (3.92)	235 (5.79)	63 (5.66)	79 (1.25)	96 (1.58)	28 (1.89)	0.89
2	Butt	191 (4.14)	120 (2.95)	35 (3.14)	329 (5.19)	304 (5.00)	78 (5.26)	0.54
3	Barnholtz-Sloan	765 (16.58)	906 (22.30)	291 (26.15)	691 (10.91)	817 (13.44)	268 (18.08)	0.30
4	Quaye	767 (16.62)	544 (13.39)	149 (13.39)	1118 (17.65)	1098 (18.07)	247 (16.67)	0.34
5	Song	2619 (56.75)	2192 (53.96)	555 (49.87)	4029 (63.60)	3667 (60.33)	842 (56.82)	0.86
6	Verhaegh	92 (1.99)	65 (1.60)	20 (1.80)	89 (1.40)	96 (1.58)	19 (1.28)	0.34

[a]Hardy-Weinberg equilibrium (HWE) in control subjects.

Supplementary Table S3: Distributions of the genotypes and alleles of the *H19* rs2839698 polymorphism

Number	First Author	Frequency distributions of the genotypes						HWE[a]
		Case			Control			
		GG N (%)	GA N (%)	AA N (%)	GG N (%)	GA N (%)	AA N (%)	
1	Hua	552 (32.94)	418 (30.53)	79 (24.38)	729 (39.84)	565 (40.53)	103 (36.01)	0.65
2	Li	583 (34.79)	462 (33.75)	102 (31.48)	666 (36.39)	462 (33.14)	75 (26.22)	0.67
3	Gong	237 (14.14)	220 (16.07)	39 (12.04)	99 (5.41)	80 (5.74)	27 (9.44)	0.10
4	Yang	250 (14.92)	195 (14.24)	55 (16.98)	284 (15.52)	178 (12.77)	38 (13.29)	0.18
5	Verhaegh	54 (3.22)	74 (5.41)	49 (15.12)	52 (2.84)	109 (7.82)	43 (15.03)	0.31

[a]Hardy-Weinberg equilibrium (HWE) in control subjects.

Supplementary Table S4: Distributions of the genotypes and alleles of the *H19* rs217727 polymorphism

Number	First Author	Frequency distributions of the genotypes						HWE[a]
		Case			Control			
		GG N (%)	GA N (%)	AA N (%)	GG N (%)	GA N (%)	AA N (%)	
1	Hua	431 (32.04)	467 (32.25)	148 (27.36)	573 (38.82)	665 (37.55)	156 (29.94)	0.07
2	Li	480 (35.69)	514 (35.50)	153 (28.28)	456 (30.89)	570 (32.19)	177 (33.97)	0.96
3	Xia	160 (11.90)	156 (10.77)	148 (27.36)	139 (9.42)	212 (11.97)	116 (22.26)	0.05
4	Yang	160 (11.90)	252 (17.40)	88 (16.27)	193 (13.08)	244 (13.78)	63 (12.09)	0.30
5	Verhaegh	114 (8.48)	59 (4.07)	4 (0.74)	115 (7.79)	80 (4.52)	9 (1.73)	0.29

[a]Hardy-Weinberg equilibrium (HWE) in control subjects.

Supplementary Table S5: Sensitivity analysis of rs217727 in dominant model

Study omitted	Cancer type	OR (95%CI)	*P*	*P* for heterogeneity	I^2
Hua (2016)	bladder	0.91 (0.71–1.18)	0.478	0.011	73.00%
Li (2016)	colorectal	0.96 (0.76–1.22)	0.745	0.022	68.90%
Xia (2016)	breast	0.97 (0.78–1.19)	0.753	0.015	71.40%
Yang (2015)	gastric	0.89 (0.80–0.99)	0.025	0.290	19.90%
Verhaegh (2008)	bladder	0.97 (0.80–1.17)	0.755	0.018	70.30%

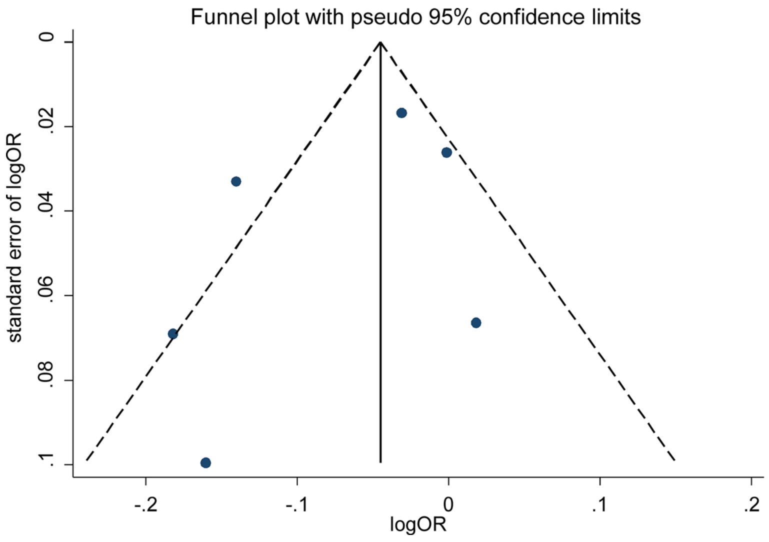

Supplementary Figure S1: Funnel plot for publication bias of the *H19* rs2107425 polymorphism and cancer risk under dominant model (Funnel plot with pseudo 95% confidence limits was used).

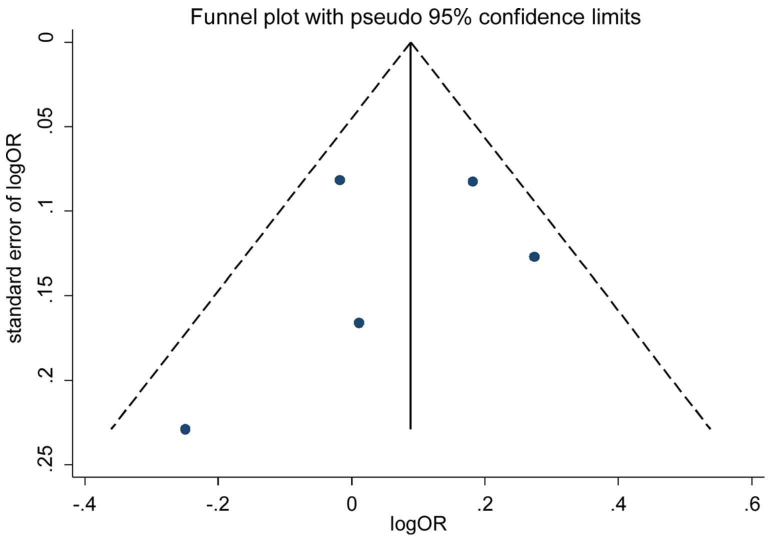

Supplementary Figure S2: Funnel plot for publication bias of the *H19* rs2839698 polymorphism and cancer risk under dominant model (Funnel plot with pseudo 95% confidence limits was used).

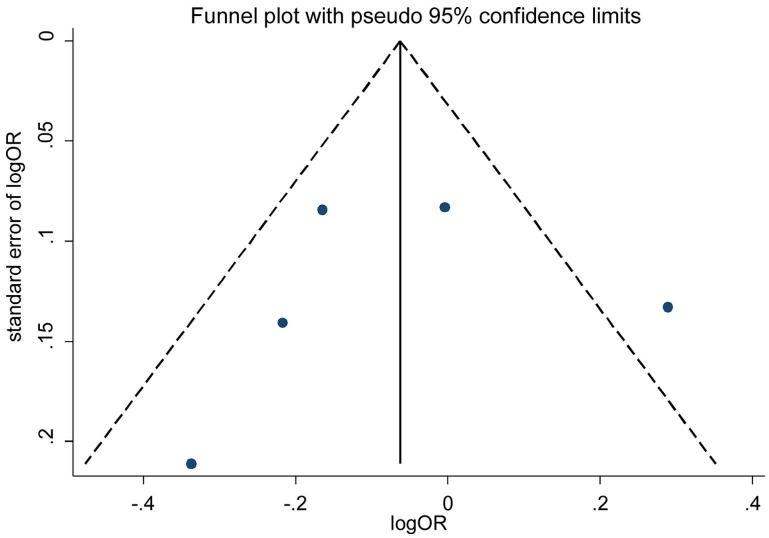

Supplementary Figure S3: Funnel plot for publication bias of the *H19* rs217727 polymorphism and cancer risk under dominant model (Funnel plot with pseudo 95% confidence limits was used).
